# Supplementary material for: Human Milk MiRNA Acts on Infections in Both the Maternal Body and the Child During Breastfeeding
Source: Life (Basel). 2026 May 25;16(6):884. doi: 10.3390/life16060884 (PMC13302718; doi:10.3390/life16060884)
Supplement: Supplementary file 1 [file life-16-00884-s001.zip › Figures S1¿CS4.pdf]

Figures S1–S4

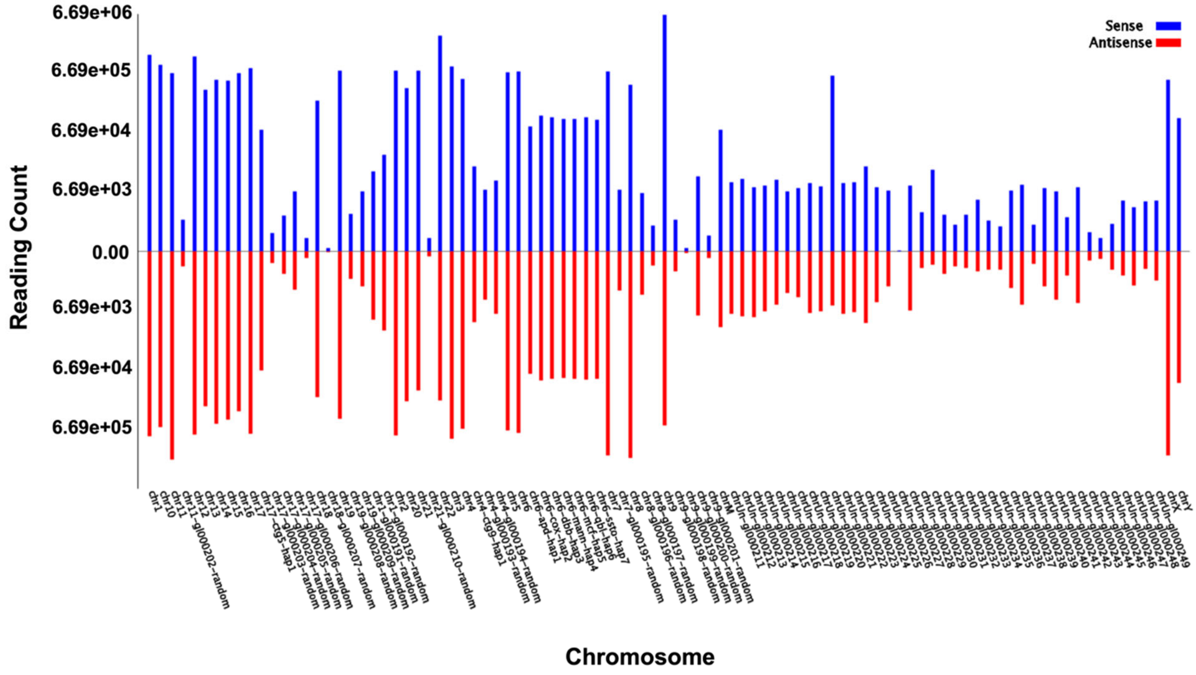

Figure S1. Mapping of the total small RNA clean reads generated from all milk samples (n=26) to the human genome. Red bars indicate the number of small RNA reads mapped on the antisense strand of the chromosome, whilst blue bars indicate to the small RNA reads number on the sense strand of the chromosome.

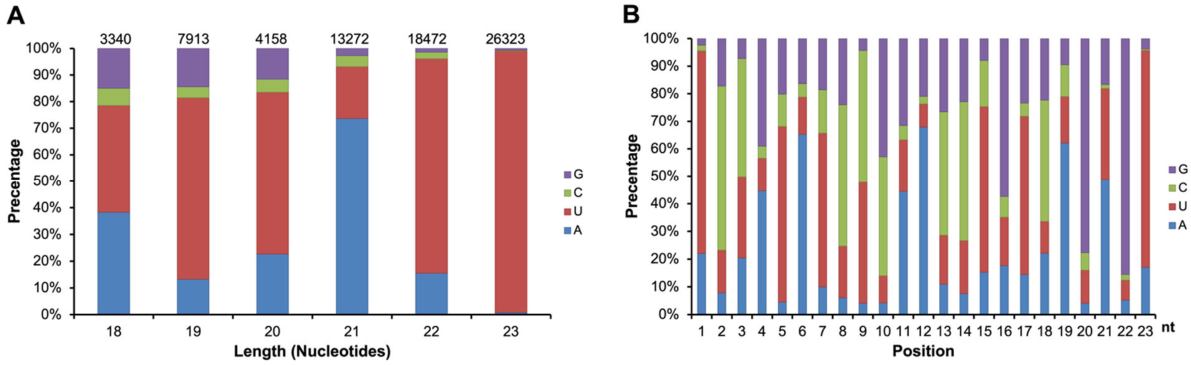

Figure S2. (A) The first nucleotide bias from the 5' end of the total clean small RNA reads (unannotated) between 18 and 23. Each color represents the four bases, and y axis indicates the percentage (%) of each base. **(B)** Base bias of all total clean small RNA reads (unannotated) at each nucleotide position, with y axis indicating the percentage (%) of each base.

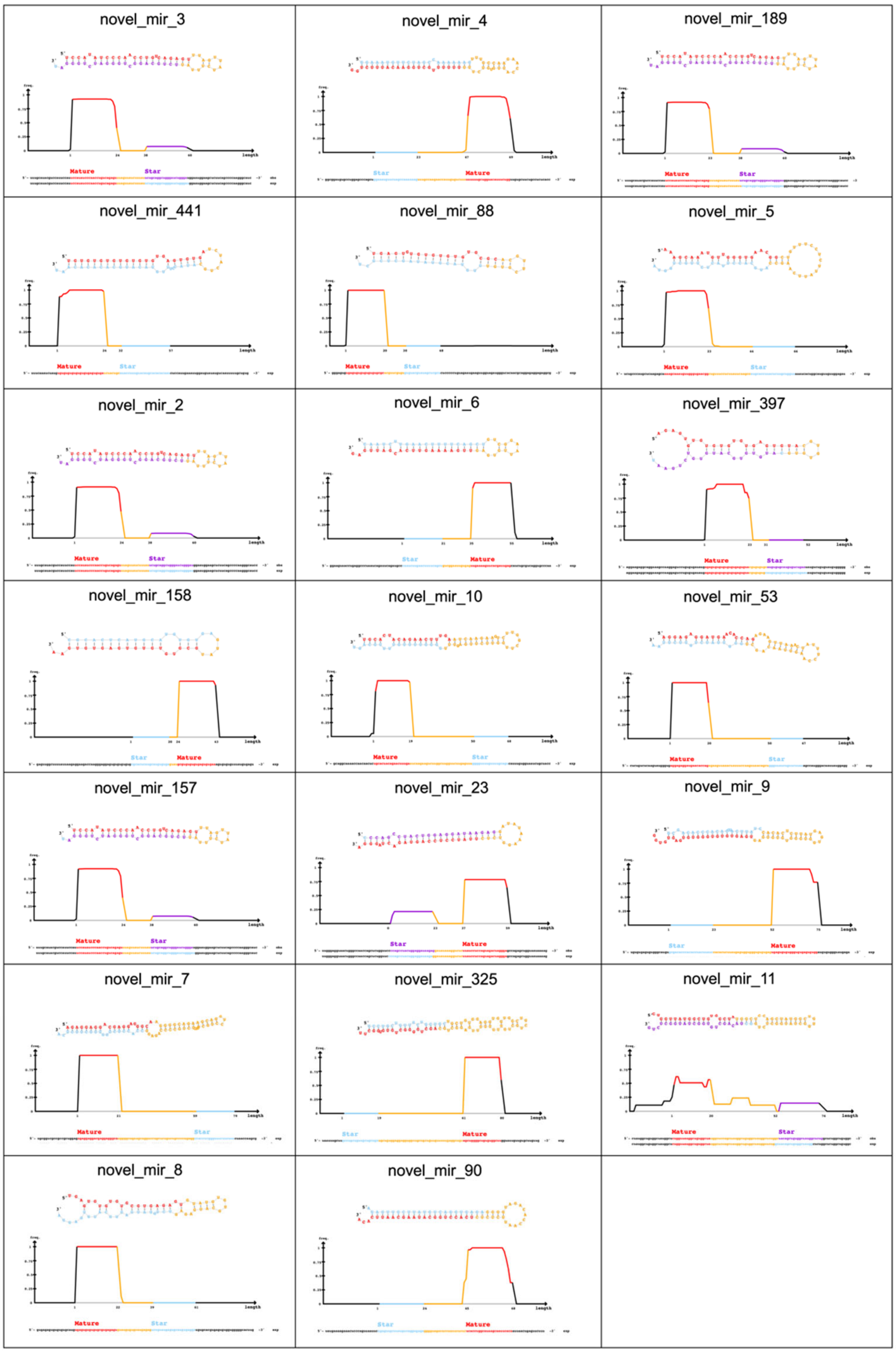

Figure S3. mirdeep analysis for the top 20 milk novel microRNAs showing their hairpin structures (stem loop) and their mature sequences.

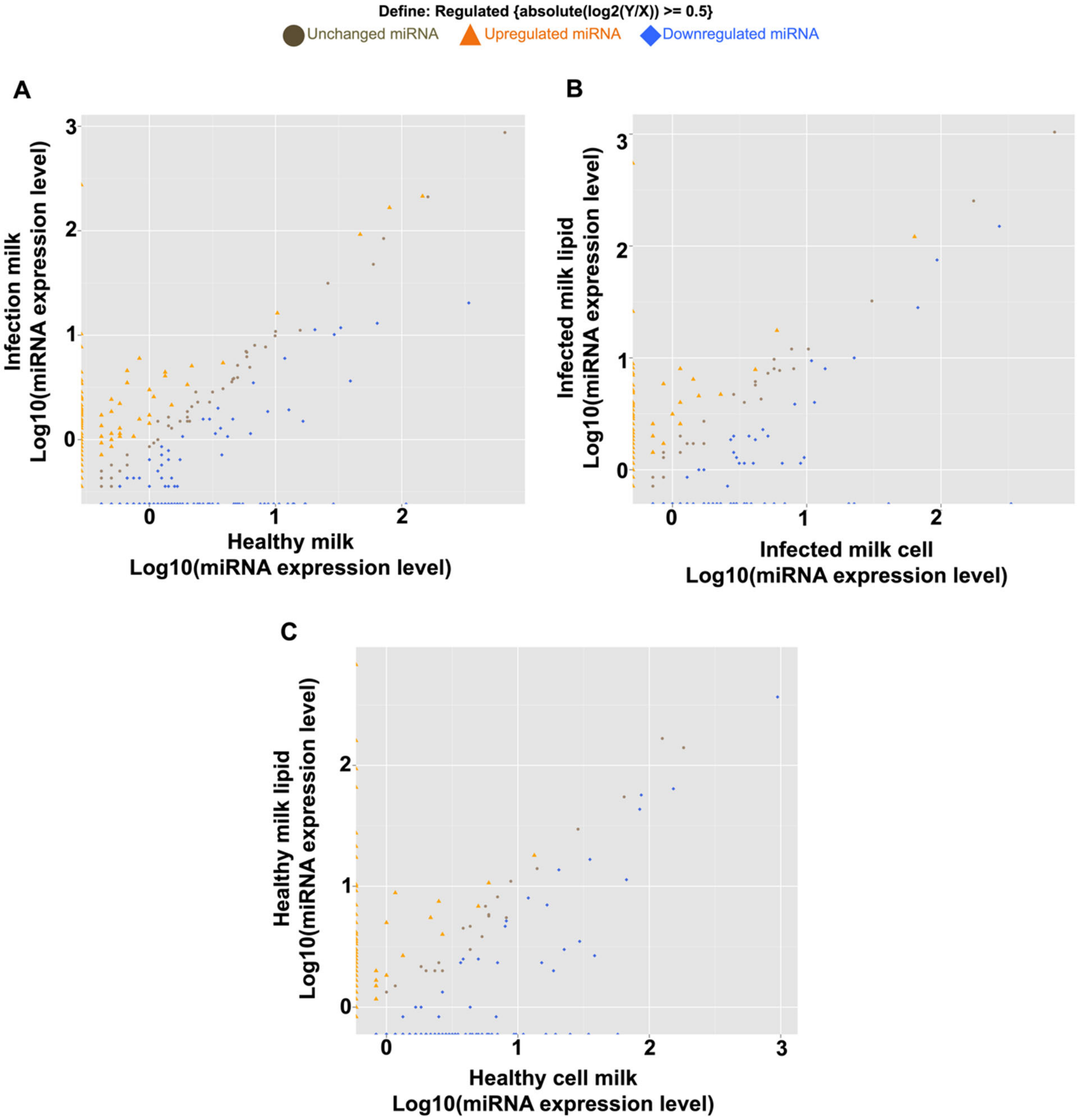

Figure S4. The differentially expression analysis of the novel microRNAs between **(A)** all combined healthy HM cells/lipids (n=14) and combined infection HM cells/lipids (n=14) sample cohorts, **(B)** between infected HM cells (n=7) and infected HM lipids (n=7), **(C)** between healthy HM cells (n=6) and healthy HM lipid (n=6).
